# Fatal outcome of posterior “reversible” encephalopathy syndrome in metastatic colorectal carcinoma after irinotecan and fluoropyrimidine chemotherapy regimen

**DOI:** 10.1186/1477-7819-12-264

**Published:** 2014-08-20

**Authors:** Natalija Dedić Plavetić, Zoran Rakušić, David Ozretić, Luka Simetić, Ana Mišir Krpan, Vesna Bišof

**Affiliations:** Department of Oncology, University Hospital Center, Kišpatićeva 12, HR-10000 Zagreb, Croatia; Department of Radiology, University Hospital Center, Kišpatićeva 12, HR-10 000 Zagreb, Croatia

**Keywords:** Chemotherapy, Fluoropyrimidine, Fluorouracil, Irinotecan, Posterior reversible encephalopathy syndrome

## Abstract

Posterior reversible encephalopathy syndrome (PRES) is a clinicoradiologic entity characterized by headaches, altered mental status, seizures, and visual disturbances. It can occur in many different clinical entities such as severe hypertension and pre-eclampsia, or due to cytotoxic or immunosuppressive therapies. The pathogenesis of PRES is unclear, with dysregulated cerebral auto-regulation and endothelial dysfunction as important mechanisms proposed. Endothelial dysfunction is important especially in cases associated with cytotoxic therapies. Herein, we describe a patient with PRES with fatal outcome, who presented 5 days after the infusion of cycle 1 of irinotecan hydrochloride, leucovorin calcium, and fluorouracil (FOLFIRI) regimen chemotherapy, without prior hypertension and other comorbidity, suggesting a link between PRES and FOLFIRI regimen. To our knowledge, this case report is the first describing PRES after FOLFIRI regimen, although others have described PRES after FOLFIRI with bevacizumab in colonic cancer patients.

## Background

Posterior reversible encephalopathy syndrome (PRES), or reversible posterior leukoencephalopathy syndrome (RPLS), is a clinicoradiologic entity characterized by headaches, altered mental status, seizures, and visual disturbances. It is associated with white matter vasogenic oedema predominately affecting the posterior occipital and parietal lobes of the brain [1].

It can occur in many different clinical entities such as severe hypertension and pre-eclampsia, or due to cytotoxic or immunosuppressive therapies, but all these heterogeneous aetiologies are grouped together because of similar neuroimaging findings. It is also often referred to as reversible posterior cerebral oedema syndrome, or hyperperfusion encephalopathy, or brain capillary leak syndrome. Nevertheless, none of these names is completely satisfactory; the syndrome is not always reversible, and it is often not confined to either the white matter or the posterior regions of the brain. The pattern of oedema in PRES can be widespread and can involve the entire brain. It was first described in 1996, when Hinchey et al. originally described a posterior hypertensive encephalopathy syndrome with a predominant posterior distribution of imaging findings on computed tomography (CT) or magnetic resonance imaging (MRI) [2].

PRES is often unsuspected by clinicians, and therefore a radiologist’s recognition of the characteristic imaging findings is key in diagnosing this syndrome. It not only helps in not delaying therapeutic measures, but also in preventing deleterious work-ups or wrong therapeutic approaches [3].

The pathogenesis of PRES is unclear, but the most prominent feature is dysregulated cerebral autoregulation and endothelial dysfunction [4]. Endothelial dysfunction is an important mechanism in the pathophysiology of PRES, especially in cases associated with cytotoxic therapies or preeclampsia [3]. The pathogenesis of PRES is controversial. One theory postulates injury to capillary bed secondary to hypertension, leading to hyperperfusion and cerebral oedema. However, the theory fails to explain both the absence of underlying hypertension in up to 20 to 30% of patients and the lack of a positive correlation between cerebral oedema and severity of hypertension. Another hypothesis is that vasoconstriction in response to evolving hypertension leads to reduced brain perfusion and cerebral oedema [5, 6]. Cytotoxic drugs have direct toxic effects on vascular endothelial cells, leading to disrupted blood-brain barrier, consequent capillary leakage, and vasogenic oedema.

Common neuroradiological findings include scattered hypodense regions involving bilateral cerebral hemispheres on multislice computed tomography (MSCT) scans in up to half of the cases. Differential diagnosis includes many conditions such as stroke, vasculitis, encephalitis, venous thrombosis, toxic or metabolic encephalopathy, and demyelinating disorders, among others.

Fatal outcome may result from increased intracranial pressure, mostly due to progressive cerebral oedema, intracerebral haemorrhage, or as a complication of the underlying conditions. However, one of the largest case series reported highlights the potential grave consequences of this disorder; among 22 patients studied, 6 died and many survivors had permanent neurologic disability [7].

Herein, we describe the case of a patient with PRES who presented 5 days after the infusion of cycle 1 of irinotecan hydrochloride, leucovorin calcium, and fluorouracil (FOLFIRI) regimen chemotherapy, suggesting a link between PRES and FOLFIRI regimen.

## Case presentation

**Figure 1:**
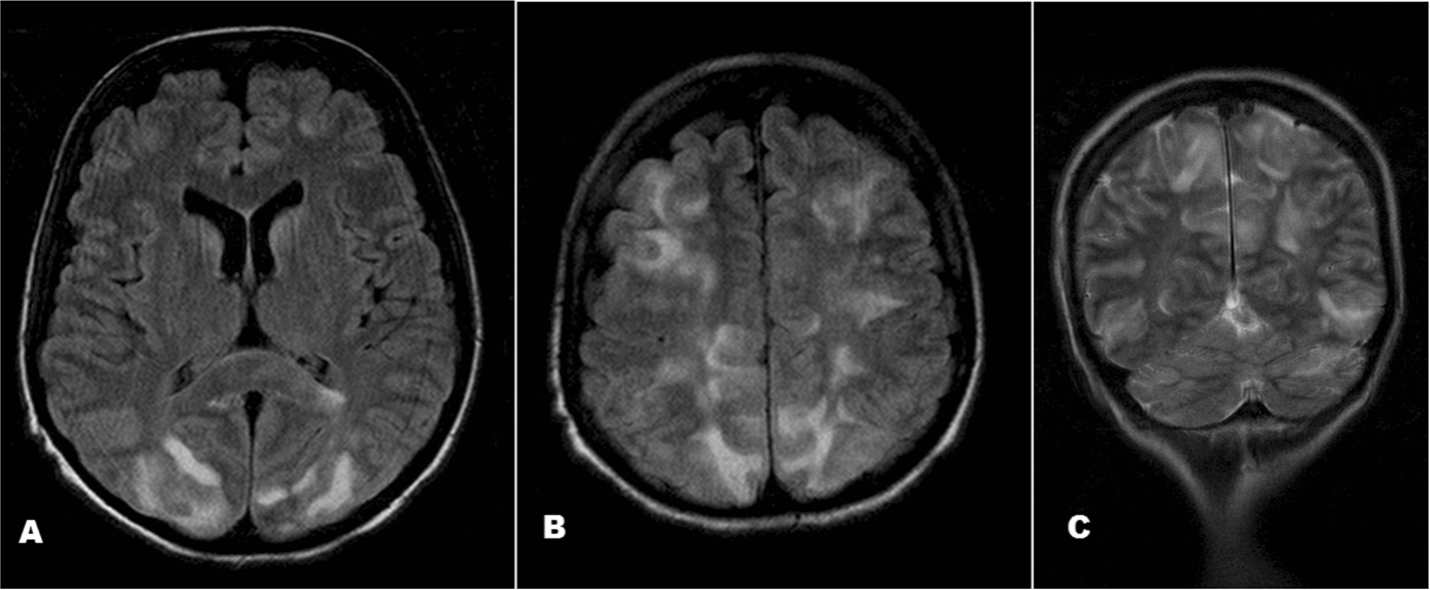
**MRI brain images, axial and coronal. (A and** **B)** Axial FLAIR images show bilateral cortical/subcortical hyperintense lesions involving occipital lobes and frontal and parietal watershed zines-typical findings in PRES. **(C)** Coronal T2W image demonstrating predilection of PRES for posterior circulation-bilateral, almost symmetric areas of oedema in parietal and occipital lobes and cerebellar hemispheres.

A 45-year-old woman diagnosed with stage IV metastatic colorectal cancer one month previous, was admitted to hospital due loss of consciousness after a *grand mal* seizure attack; she had had no previous seizures. Medical history revealed conisation due to cervical intraepithelial neoplasia CIN III at the age of 26, with regular gynaecologic follow-up visits afterwards. Familiar history revealed the mother diagnosed with endometrial cancer, father had died due to colonic carcinoma, and grandfather had died due to urinary bladder carcinoma. At the age of 45 she presented with an acute abdomen and underwent urgent surgery due to perforated carcinoma of the sigmoid colon. A Hartmann operation was performed and several hepatic metastases were found as well as an osteolytic lesion of the right acetabulum which was conformally irradiated before starting chemotherapy. Palliative irradiation with a total dose of 20 Gy on the right hip was performed. Five days before admission, cycle 1 of FOLFIRI chemotherapeutic regimen was started. The patient’s performance status before chemotherapy was excellent and without any signs of infection and/or sepsis. Dosing consisted of irinotecan, 180 mg/m^2^; leucovorin, 400 mg/m^2^ (both in 2 hours infusion); fluorouracil, 400 mg/m^2^ (intravenous push) on Day 1 of cycle 1; and fluorouracil, 2,400 mg/m^2^ (48 hours infusion) during Day 1 and Day 2.On Day 5 after FOLFIRI administration the patient awoke with a headache and after a few minutes experienced two episodes of generalized tonic-clonic seizures and was urgently admitted to hospital. She had no focal neurologic deficit. The patient’s blood pressure during hospitalization was mildly elevated (systolic blood pressure range 140 to 150 mm Hg, diastolic 100 to 105). MSCT contrast enhanced scan was performed on admission and revealed hypodense areas in subcortical white matter parasagitally in parieto-occipital region bilaterally. Identical, but more discrete changes were found bilaterally in the frontal region. There were no metastatic lesions, hydrocephalus, or intracranial haemorrhage. MSCT finding was suspicious of PRES and MRI of the brain was performed with Siemens Magnetom Symphony (Siemens, Erlangen, Germany). Contrast media was injected intravenously. MRI revealed patchy areas of increased fluid attenuated inversion recovery signal intensity in the occipital and posterior parietal lobes, consistent with RPLS (Figure 1).

After two days of intensive therapy consisting of gradual blood pressure control and anti-oedematous therapy with 100 mL of 20% mannitol intravenously four times daily, and levetiracetam 500 mg intravenously twice a day, the patient fell into a coma and died a few hours later. Autopsy revealed lung and liver metastases, and prominent cerebral oedema with enlarged perivascular and pericellular spaces. There was focal subarachnoidal haemorrhage in the left frontoparietal region, without blood penetration into cerebral parenchyma. There was no cerebral infection on autopsy specimens. Other possible causes, such as progressive multifocal leukoencephalopathy, were also considered but ruled out clinically and radiologically.

## Conclusions

To our knowledge, this case report is the first describing PRES after FOLFIRI regimen, although others have described PRES after FOLFIRI with bevacizumab in colonic cancer patients [8–10] and breast cancer patients [11]. It is not surprising that bevacizumab is related with PRES due to its mechanism of action and vasculature being involved in pathogenesis. Our patient experienced fatal outcome PRES after only one cycle of FOLFIRI chemotherapy regimen, and without prior hypertension and other comorbidity. A relative paucity of sympathetic adrenergic innervation to the vertebral basilar system is suggested to account for the tendency for this syndrome to involve structures supplied by the posterior circulation [12]. There were no other predisposing factors consistent with this proposed mechanism of vascular dysregulation such as those described by Tam et al. [13], who regard patients with significant fluid overload (more than 10% of the baseline weight), a mean blood pressure higher than 25% of baseline, and a creatinine level greater than 1.8 mg/dL (160 μmol/L) to be high risk for RPLS. Although our patient had a mean blood pressure higher than 25% of baseline, she was not otherwise at high risk for the development of PRES. She was not hypervolemic and her serum creatinine level was normal. Former palliative radiotherapy of right acetabulum with only 20 Gy total dose was not likely to cause clinically relevant immune-compromised status. Therefore, we did not find it as a significant possible cause of PRES in our patient. Despite sigmoid colon perforation, there were no sepsis and abdominal infection which might predispose the patient to PRES. Direct causal relationships between chemotherapy agents and PRES have been difficult to establish, and the correlation is more common with cyclosporine and tacrolimus. Other associated agents include methotrexate, the alpha interferons, cisplatin, cytarabine, cyclophosphamide, doxorubicin hydrochloride (Adriamycin), vincristine sulfate (Oncovin), and prednisone, or combinations involving doxorubicin, ifosfamide, and etoposide.

The pathophysiology linking fluoropyrimidines like fluorouracil has not been well described, but several pathogenic explanations have been suggested. The role of fluoropyrimidines and oxaliplatin in PRES has been described in Femia’s et al. case report and literature review [14].

The cerebral toxicity of fluorouracil, although rare, is well described as a multifocal inflammatory leukoencephalopathy [15, 16]. Multifocal inflammatory leukoencephalopathy typically develops from 6 weeks to 5 months after the initiation of combination chemotherapy with levamisole hydrochloride and fluorouracil. Encephalopathy in our patient developed only five days after chemotherapy administration. Multifocal inflammatory leukoencephalopathy is a clinically and radiographically distinct syndrome from PRES. To our knowledge, no reports in the literature associate irinotecan, a type I topoisomerase inhibitor, with the development of PRES or other encephalopathy syndromes. Leucovorin, a derivative of folic acid, would not be expected to contribute to the development of PRES. Several other investigators observed an association between PRES and bevacizumab administration [10, 17]. Toxic damage to the vascular endothelium or blood-brain barrier caused by immunosuppressant and cytotoxic medications is further postulated to contribute to the pathophysiological features of PRES [18]. In this case, we could point to either of the cytotoxic agents fluorouracil or irinotecan. CT may show scattered hypodense regions involving bilateral cerebral hemispheres in up to half of the cases [19, 20]. In our patient, hypodense areas in subcortical white matter parasagitally in parieto-occipital region bilaterally were found on CT imaging.

Furthermore, haemorrhage is increasingly being recognised in PRES and does not exclude the diagnosis [21]. This can be anatomically located in the brain parenchyma as a focal haematoma or petechial gyral pattern, or in the subarachnoid space. Intracranial haemorrhage is known to occur in PRES with an incidence of approximately 15%, range 10 to 17% in different studies [22]. The pathological mechanism is still not well understood but is thought to relate to hypertension and hyperperfusion or vasculopathy with resulting hypoperfusion. Varying patterns of haemorrhage have been identified, including large focal haematomas, subarachnoid haemorrhage (like in our patient), or multiple minute foci of haemorrhage [23].

Prompt recognition of this syndrome will allow initiation of immediate treatment and appropriate alterations in chemotherapy regimens. Clinical suspicion with abrupt onset of symptoms in certain subsets of susceptible patients and imaging, especially MRI, plays an essential role in diagnosing this condition that radiologists and clinicians should be well aware of.

## Consent

Written informed consent was obtained from the next-of-kin of the patient for publication of this case report and any accompanying images. A copy of the written consent is available for review by the Editor-in-Chief of this journal.

## Abbreviations

CT: Computed tomography
FOLFIRI: Irinotecan hydrochloride, leucovorin calcium, and fluorouracil
MRI: Magnetic resonance imaging
MSCT: Multislice computed tomography
PRES: Posterior reversible encephalopathy syndrome
RPLS: Reversible posterior leukoencephalopathy syndrome.
